# Effectiveness of Teleretinal Imaging–Based Hospital Referral Compared With Universal Referral in Identifying Diabetic Retinopathy: A Cluster Randomized Clinical Trial

**DOI:** 10.1001/jamaophthalmol.2019.1070

**Published:** 2019-05-09

**Authors:** Sanil Joseph, Ramasamy Kim, Ravilla D. Ravindran, Astrid E. Fletcher, Thulasiraj D. Ravilla

**Affiliations:** 1 Lions Aravind Institute of Community Ophthalmology, Aravind Eye Care System, Madurai, India; 2 Aravind Eye Care System, Madurai, India; 3 Faculty of Epidemiology & Population Health, London School of Hygiene & Tropical Medicine, London, United Kingdom

## Abstract

**Importance:**

Studies in high-income countries provide limited evidence from randomized clinical trials on the benefits of teleretinal screening to identify diabetic retinopathy (DR).

**Objective:**

To evaluate the effectiveness of teleretinal-screening hospital referral (TR) compared with universal hospital referral (UR) in people with diabetes.

**Design, Setting, and Participants:**

A cluster randomized clinical trial of 8 diabetes clinics within 10 km from Aravind Eye Hospital (AEH), Madurai, India, was conducted. Participants included 801 patients older than 50 years. The study was conducted from May 21, 2014, to February 7, 2015; data analysis was performed from March 12 to June 16, 2015.

**Interventions:**

In the TR cohort, nonmydriatic, 3-field, 45° retinal images were remotely graded by a retinal specialist and patients with DR, probable DR, or ungradable images were referred to AEH for a retinal examination. In the UR cohort, all patients were referred for a retinal examination at AEH.

**Main Outcomes and Measures:**

Hospital-diagnosed DR.

**Results:**

Of the 801 participants, 401 were women (50.1%) (mean [SD] age, 60.0 [7.3] years); mean diabetes duration was 8.6 (6.6) years. In the TR cohort, 96 of 398 patients (24.1%) who underwent teleretinal imaging were referred with probable DR (53 [13.3%]) or nongradable images (43 [10.8%]). Hospital attendance at AEH was proportionately higher with TR (54 of 96 referred [56.3%]) compared with UR (150 of 400 referred [37.5%]). The intention-to-treat analysis based on all patients eligible for referral in each arm showed that proportionately more patients with TR (36 of 96 [37.5]%) were diagnosed with DR compared with UR (50 of 400 [12.5%]) (unadjusted risk ratio [RR], 3.00; 95% CI, 2.01-4.48). These results were little changed by inclusion of covariates (RR, 2.72; 95% CI, 1.90-3.91). The RR was lower in the per-protocol analysis based on all patients who adhered to referral (covariate-adjusted RR, 1.75; 95% CI, 1.12-2.74). Diagnoses of DR were predominantly mild or moderate nonproliferative DR (36 in TR and 43 in UR). In the UR arm, there were 4 cases of severe nonproliferative DR and 2 cases of proliferative DR. Age (RR, 0.98; 95% CI, 0.95-0.99), female sex (RR, 0.79; 95% CI, 0.64-0.98), and hypertension diagnosis (RR, 0.81; 95% CI, 0.68-0.95) were factors associated with lower attendance. Those with higher secondary educational level or more were twice as likely to attend (RR, 2.00; 95% CI, 1.32-3.03).

**Conclusions and Relevance:**

The proportionate yield of DR cases was higher in the TR arm, confirming the potential benefit, at least in the setting of eye hospitals in India, of a targeted referral approach using teleretinal screening to identify patients with DR.

**Trial Registration:**

ClinicalTrials.gov identifier: NCT02085681

## Key Points

**Question:**

Does screening for diabetic retinopathy by teleretinal imaging in physician offices in India lead to higher adherence to eye hospital referral and a greater yield of diabetic retinopathy cases compared with a strategy of referral of all eligible patients with diabetes?

**Findings:**

In a cluster randomized clinical trial of 801 patients with diabetes, proportionately more patients in the teleretinal group attended the hospital eye examination and had confirmed diabetic retinopathy compared with the control group.

**Meaning:**

The results suggest that, in the Indian setting, teleretinal screening is an effective approach for identifying diabetic retinopathy.

## Teaser

This cluster randomized clinical trial evaluates the use of teleretinal imaging in diabetes clinics to identify diabetic retinopathy and refer patients for hospital care.

## Introduction

It has been estimated that there are 65 million people with diabetes in India, and another 22 million in the high-risk prediabetes stage, with the total number of diabetes cases projected to increase to 109 million by 2035.^1^ These numbers suggest a great need to expand services for diabetes and develop appropriate prevention and control interventions.^2^ A visual consequence of diabetes is diabetic retinopathy (DR). Population-based surveys in India have reported DR in 10% to 30% of adults with diabetes, with higher rates found in older people and urban areas.^3,4,5,6,7,8,9,10^ Early identification of DR with appropriate treatment is necessary to reduce the rate of vision loss. Key recommendations from the World Health Organization include periodic eye examinations, strategies to increase awareness among people with diabetes and their physicians of the importance of eye examinations, and methods to address barriers to the uptake of the strategies.^2^ In India, ad hoc one-time screening by outreach camps in rural or urban areas has been undertaken^11^ but does not fulfill the requirement for periodic eye examinations. Even specialist diabetes clinics face barriers owing to lack of awareness of DR by patients and paramedical staff and low uptake of retinal specialist referral because of costs and limited access.^12,13^ In developing countries, including India, inadequate eye care resources, both in infrastructure and personnel, is a reality.^14^ Teleretinal screening for DR is increasingly being used in India.^15,16^ There is limited evidence from randomized clinical trials on the benefits of teleretinal screening from studies in high-income countries.^17,18,19,20,21^ The objective of the present study was to evaluate the effectiveness of teleretinal screening in the Indian setting.

## Methods

### Study Design

We conducted a cluster randomized clinical trial with the primary outcome of hospital-confirmed DR. We investigated factors associated with hospital attendance as a secondary outcome. The unit of randomization was a diabetes center, and the centers were randomized to teleretinal imaging and hospital referral (TR) or universal hospital referral (UR). A cluster randomized clinical trial was required to avoid patients in the same center being offered different referral interventions, which might present logistical and ethical difficulties.

The protocol (Supplement 1), including the procedures for fully informed consent, was approved by the institutional review board of Aravind Eye Hospital (AEH), Madurai, India, and the London School of Hygiene & Tropical Medicine, London, United Kingdom, and complied with the Declaration of Helsinki.^22^ Eligible participants in the TR arm were given an information sheet in the local language that explained the purpose of the study and procedures involved and asked for consent for fundus imaging and access to their clinical records. Patients in the UR arm consented to access of their medical records. Patients were reassured about the confidentiality of their personal and clinical details and, in the TR arm, that their usual care would not be affected by nonparticipation. The information was read to patients who were illiterate. All participants were given the information sheet to take home and the contact number of the study coordinator. Written consent or, for illiterate patients, a thumb impression, was obtained. A data safety and monitoring committee was established to oversee study procedures and safety aspects. The patients did not receive financial compensation.

Within each center, there was no allocation of individuals to interventions. We recruited centers located 10 km or less from AEH to minimize barriers due to distance and cover the most densely populated (<5 km) and outer periurban/rural areas. Centers were randomly and equally balanced across the 2 arms by 2 strata: distance less than 5 km and 5 to 10 km or more. Centers were private diabetes clinics (3 attached to medium-sized general hospitals) and staffed by 1 physician or diabetologist with nursing and administrative support. Such clinics typically provide care for 15 to 20 new or returning people with diabetes at a clinic session and do not have a systematic method of referring patients for eye examination. We identified and shortlisted 16 diabetes clinics that met the distance criteria and finalized 8 clinics based on acceptance and availability of suitable space.

We recruited university graduates to be study coordinators in each clinic. All coordinators underwent a structured training program in diabetes and DR, including patient counseling (diabetes control, awareness of vision problems, and need for regular eye examination). In both arms, the counseling and awareness creation materials were standardized. The study was conducted from May 21, 2014, to February 7, 2015; data analysis was performed from March 12 to June 16, 2015.

### Inclusion and Exclusion Criteria

Inclusion criteria were diagnosis of diabetes as designated by *International Classification of Diseases, Ninth Revision* code 250 in patients 50 years or older. Patients were excluded if they were younger than 50 years, had been screened for DR in free camps organized by a diabetes center, had undergone retinal examination in the previous year, or had any disability (physical or mental) that prevented them traveling to the eye hospital.

### Study Procedures

All patients in either arm meeting the inclusion and exclusion criteria were referred by the registration staff at each center to the study coordinator who sought full informed patient consent prior to enrollment. Those enrolled provided information on their sociodemographic characteristics, duration of diabetes, complications, and treatment. The diagnosis of hypertension was obtained from the clinical history. In each clinic, participants were counseled about diabetes and the importance of vision screening. In the TR arm, the diabetes center was equipped with a handheld, nonmydriatic fundus camera (Smartscope PRO software, version 3.2.6.3498; Optomed Oy Ltd).

### Study Arms

In the TR arm of the trial, the study coordinator in each clinic was trained in fundus photography through a structured 2-week training session. Patients at the clinics underwent nonmydriatic, 3-field, 45° retinal imaging and the images were transferred to AEH via internet using the Aravind Diabetic Retinopathy Evaluation Software that enabled a retinal specialist to read and grade the image and send feedback immediately to the diabetes clinics. Patients with DR or probable DR or ungradable image were counseled about the risk of vision loss and referred to AEH. This procedure reflects a category 1 program for DR as defined by the American Telemedicine Association.^23^ Eligible patients in the UR arm were given standard counseling and referred to AEH. In both arms, each referred patient was given a referral card with the provisional appointment date and contact details of the hospital appointment clerk.

The study was carried out for 6 months and a window of 1 month after referral was given for the patients to visit the eye hospital. Up to 3 motivating telephone reminders were made for nonattenders. For all final nonattenders, information was sought on their reason for nonattendance and possible receipt of care at other eye care professional settings and any DR findings. The AEH retinal specialists were masked to study arm and the teleretinal findings. Diabetic retinopathy was graded using the international classification of DR by the American Academy of Ophthalmology guidelines.^24^

### Sample Size

We estimated a total sample size of 612 patients equally distributed in each arm based on an estimated DR proportion of 30% in UR referrals compared with 50% in TR at a power of 90%, α level of .01, and design effect of 2. These assumptions of the proportion of DR in people with diabetes in the UR arm were based on estimates from previous studies in India and for an urban and older age group.^3,4,6^ We assumed a 50% proportion of DR in those referred by TR as having probable DR. We used a conservative design effect of 2 available from a previous study.^3^

### Statistical Analysis

We used Poisson regression for the primary outcome analysis comparing DR in the TR arm with that in the UR arm and reported the risk ratio (RR) and 95% CI in unadjusted analysis and covariate-adjusted analysis. We report 2 strategies of analysis: the primary intention-to-treat analysis including all eligible patients in each randomized arm irrespective of adherence to referral and the secondary per-protocol analysis including patients who adhered to referral (defined as attendance at AEH or other eye specialist). We used Stata, version 14.0 (StataCorp) and took account of the cluster design by use of the survey commands (svy) in Stata. The exception was the per-protocol primary outcome analysis where the numbers were too small for reliable survey adjustment. We performed paired, 2-tailed testing with the level of significance set as *P* < .05.

## Results

We invited 13 clinics that met the distance criteria for participation; 8 clinics accepted (eFigure in Supplement 2). These clinics were randomized to TR (n = 4) or UR (n = 4) equally stratified by the distance criteria. Of 860 patients invited to take part in the trial, 23 patients refused and 46 were not eligible (Figure). We finished recruitment in each clinic when they attained 100 patients as this was more than sufficient for our sample size calculation. There was no significant difference between the arms in baseline demographic or clinical variables except for income level, which was higher in the TR cohort (Table 1). The mean (SD) diabetes duration was 8.6 (6.6) years and similar in both arms; 190 patients (23.7%) were receiving insulin treatment. Hemoglobin A_1c_ measurements were available only in 133 participants in the TR and 5 in the UR arms and were not included in the analysis. Three patients in the TR cohort refused retinal imaging, 301 patients (75.6%) had no signs of DR, and 53 patients (13.3%) had some signs, the majority being nonproliferative DR (NPDR): mild (33) moderate (8), and severe (4). Six patients (4.5%) were graded as having proliferative DR (PDR), either high risk (3) or advanced (3); 2 people had diabetic macular edema with mild NPDR. Images could not be graded in 43 patients (10.8%) mainly because of narrow aperture (28). These patients were also referred to AEH, making a total of 96 participants with TR. Attendance at AEH as a proportion of those referred was higher in the TR arm (54 [56.3%]) compared with the UR arm (150 [38%]) (*P* = .01). In both arms the main reason for nonattendance was that the patient was not willing or could not be contacted by the study coordinator (Figure). A small number of patients (14) stated they were visiting other ophthalmologists but we were unable to obtain their clinical findings. In patients who underwent AEH retinal examination, the evaluation was incomplete in 2 patients in the UR and 1 patient in the TR group (patients prematurely leaving the hospital). Diabetic retinopathy was diagnosed in 36 of 53 examined patients (67.9%) in the TR arm and 50 of 148 patients (33.8%) in the UR arm. Diabetic retinopathy diagnoses were predominantly mild or moderate NPDR (36 in TR and 43 in UR). In the UR arm, there were 4 cases of severe NDPR and 2 cases of PDR and none within these categories in the TR arm.

**Figure.**
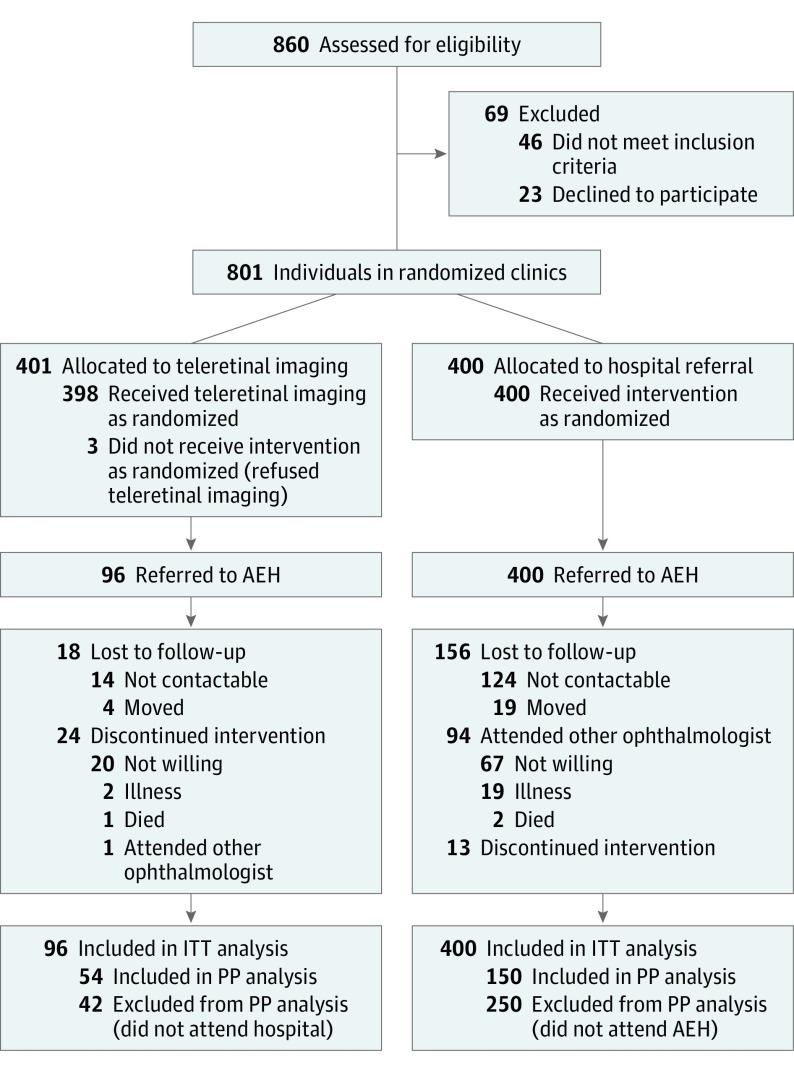
Flowchart of Randomized Individuals AEH indicates Aravind Eye Hospital; ITT, intention-to-treat; PP, per protocol.

**Table 1.** Baseline Characteristics by Randomized Study Arm

Variable	Study Arm, No. (%)	
	TR (n = 401)	UR (n = 400)
Age, mean (SD), y	59.9 (7.1)	60.2 (7.5)
Women	197 (49.1)	204 (51.0)
Distance, km		
0 to <5	78 (19.5)	33 (8.3)
≥5 to <10	76 (19.0)	74 (18.5)
≥10	247 (61.6)	293 (73.3)
Income, %^a^		
≤120 000 INR	235 (58.6)	335 (83.8)
>120 000 INR	166 (41.4)	65 (16.3)
Educational level		
No education	59 (14.7)	49 (12.3)
Primary schooling	60 (15.0)	81 (20.3)
Secondary schooling	184 (45.9)	181 (45.3)
Higher secondary and above	98 (24.4)	89 (22.3)
Blood glucose, mean (SD), mg/dL	222.8 (78.2)	210.5 (85.8)
Diabetes duration, mean (SD), y	8.4 (6.5)	8.8 (6.6)
Insulin treatment	90 (22.4)	100 (25.0)
Cardiovascular disease	22 (5.5)	21 (5.3)
Hypertension	131 (32.7)	142 (35.5)

Abbreviations: INR, Indian rupee; TR, teleretinal-screening hospital referral; UR, universal hospital referral.

SI conversion: To convert glucose to millimoles per liter, multiply by 0.0555.

^a^ 120 000 INR is equivalent to approximately US $1700.

The intention-to-treat results showed that more patients in the TR cohort (36 of 96 [37.5%]) were diagnosed with DR compared with the number in the UR cohort (50 of 400 [12.5%]) (RR, 3.00; 95% CI, 2.01-4.48) (Table 2). Inclusion of baseline covariates made little change to the RR but improved the precision of the estimate (RR, 2.72; 95% CI, 1.90-3.91). Covariates associated with a higher risk of DR were duration of diabetes and insulin treatment. In the per-protocol analysis including only patients who adhered to referral, DR was twice as common in the TR arm (unadjusted RR, 2.00; 95% CI, 1.30-3.07 and covariate-adjusted RR, 1.75; 95% CI, 1.12-2.74) (eTable in Supplement 2). Diabetes duration was associated with a higher risk of DR (adjusted RR, 1.05; 95% CI, 1.02-1.08).

**Table 2.** Hospital-Diagnosed Diabetic Retinopathy by Randomized Arm in Intention-to-Treat Analysis^a^

Diabetic Retinopathy^b^	Risk Ratio (95% CI)^c^	*P* Value^c^
TR vs UR^d^	3.00 (2.01-4.48)	<.001
TR vs UR^e^	2.72 (1.90-3.91)	<.001
Age	0.96 (0.92-1.01)	.13
Women	0.71 (0.50-1.01)	.06
Blood glucose	1.00 (0.99-1.00)	.78
Diabetes duration	1.05 (1.02-1.08)	.01
Insulin treatment	1.77 (1.05-2.98)	.04
Cardiovascular disease	0.71 (0.33-1.52)	.32
Hypertension	0.79 (0.49-1.26)	.27

Abbreviations: TR, teleretinal-screening hospital referral; UR, universal hospital referral.

^a^ All patients referred to hospital in each randomized arm regardless of adherence to referral.

^b^ Hospital diagnosed.

^c^ Determined through design-adjusted Poisson model.

^d^ Unadjusted for other covariates

^e^ Adjusted for other covariates.

Of the 401 patients recruited in the TR arm, 43 participants (10.7%) had ungradadable images; this was 44.8% of the patients referred to AEH (n = 96). In the 54 cases of DR identified by retinal imaging who attended AEH, 36 cases (66.7%) were confirmed by retinal examination. Patients not found to have DR had a teleretinal grading of mild NPDR (5), severe NPDR (1), or diabetic macular edema (1); 11 images (20.4%) were ungradable. Of the 53 patients who were referred with clear DR grading, 43 patients (81.1%) attended AEH. Teleretinal grades for the 42 patients who failed to attend AEH were mild NPDR (7), moderate NPDR (2), severe NPDR (1), high-risk PDR (2), advanced PDR not requiring laser therapy (3), or diabetic macular edema (1); for 26 patients (61.9%), images were nongradable.

We assessed demographic and clinical variables associated with attendance at AEH (Table 3). Patients in the TR group were 1.56 times more likely to attend than those in the UR group (95% CI, 1.26-1.93). Age (RR, 0.98; 95% CI, 0.95-0.99), female sex (RR, 0.79; 95% CI, 0.64-0.98), and hypertension diagnosis (RR, 0.81; 95% CI, 0.68-0.95) were factors associated with lower attendance, and higher secondary educational level was associated with higher attendance (2.00; 95% CI, 1.32-3.03).

**Table 3.** Factors Associated With Hospital Attendance

Attendance^a^	Risk Ratio (95% CI)^b^	*P* Value^b^
TR vs UR	1.56 (1.26-1.93)	.002
Age	0.98 (0.95-0.99)	.05
Women	0.79 (0.64-0.98)	.04
Income >120 000 INR	0.84 (0.56-1.24)	.32
Educational level		
Primary schooling	1.50 (0.79-2.86)	.18
Secondary schooling	1.40 (0.90-2.19)	.12
Higher secondary and above	2.00 (1.32-3.03)	.006
Distance >10 km	1.22 (0.93-1.60)	.13
Blood glucose	1.00 (0.99-1.00)	.59
Diabetes duration	1.01 (0.98-1.04)	.41
Insulin treatment	1.15 (0.88-1.48)	.26
Cardiovascular disease	0.95 (0.69-1.32)	.74
Hypertension	0.81 (0.68-0.95)	.02

Abbreviations: TR, teleretinal-screening hospital referral; UR, universal hospital referral.

^a^ Defined as undergoing a dilated retinal eye examination at Aravind Eye Hospital Madurai.

^b^ Determined through Poisson model adjusted for other covariates.

## Discussion

Based on our primary outcome of all patients referred according to the study protocol, we found a higher proportion of DR in the TR compared with the UR arm. In terms of the actual numbers of DR cases identified and disregarding patient and health care costs, UR might be a preferred option for identifying DR in patients with diabetes. However, taking account of actual hospital attendance, the proportionate yield of DR cases was higher in the TR arm, confirming the benefit of a targeted referral approach. We chose UR as the comparator group for ethical reasons and we facilitated their attendance by a referral card and a direct contact line at AEH. It would also be pragmatic to use the TR approach of fundus imaging and telediagnosis, given the current and growing magnitude of diabetes^25^ as well as the widespread availability of broadband and affordable imaging technologies. The strategy of referring everyone with diabetes for an annual retinal examination is unlikely to succeed in the Indian setting because of the low levels of adherence due to various barriers.^26^ Moreover, even if barriers could be addressed and adherence was increased, the enhanced demand and extra resources required would overwhelm the system. The estimated 65 million people with diabetes in India would translate to 20 to 25 additional outpatient visits each day, assuming 250 working days and 10 000 ophthalmologists capable of a detailed retinal examination. The reasons for lack of adequate eye care facilities in India for dealing with the current volume of patients include limited trained retinal specialists; diagnostic, laser, or surgical equipment; and good follow-up systems for telephone contact and recontact with patients.^14^

We found that attendance as a proportion of those referred was higher in the TR cohort. In common with other studies on health care use in India,^27^ we found that older people, women, and those with no formal education were less likely to attend the hospital. Most confirmed cases of DR were of mild or moderate NPDR. The few severe cases were in the UR arm. The major clinical risk factors for DR were duration of diabetes and receipt of insulin treatment, as have been identified in numerous studies, including those from India.^28,29^

The literature on teleretinal screening for DR is predominantly descriptive or compares preintervention and postintervention findings.^30^ In contrast, to our knowledge, there have been few randomized clinical trials, and based on Clinicaltrials.gov, none have been conducted in developing countries. Three randomized clinical trials were undertaken in the United States, with 2 in underserved settings (largely rural with high proportions of ethnic minorities)^19,20,21^ and 1 in an urban ambulatory clinic,^17^ and 1 randomized clinical trial took place in Australia.^18^ In all trials the comparator group was usual care and, additionally in the Australian trial, participants in the usual-care group were offered teleretinal screening 2 years after enrollment. In both arms patients were encouraged to obtain a full dilated eye examination. The Australian trial was a cluster randomized clinical trial in patients with no history of DR (N = 1074) in 10 general practices matched by geographic region, hospital referral pathways and size, and characteristics of patients with diabetes.^18^

The primary end points in these trials were different criteria of attendance or uptake, but none of these trials used the outcome of confirmed DR as in our study. Results in the trials favored the TR arm for attendance either for teleretinal imaging compared with dilated eye examination (59 [77% vs 14%]),^19^ receipt of dilated eye examinations (including referral following teleretinal findings (448 [87% vs 77%]),^17^ or receipt of any DR screening examination (94%; half of the examinations were both full dilated eye examination and TR imaging) compared with 56% (n = 567).^20^ In the Tribal Vision Project trial, after the usual care arm was offered teleretinal imaging, there was no difference in the proportion of any DR screening examination attendance over the 3-year follow-up.^21^ In the Australian trial, 100% screening rates were achieved in the TR arm compared with 62% in the control arm and 8.7% in the TR arm compared with 5% in the usual care arm with a final dilated eye examination diagnosis of DR.

We found that 12.5% of participants overall in the TR arm and 44.8% of those referred had ungradable images. Other trials also reported high proportions of patients in the TR arm who were referred because of poor images (86%^20^ and 36%^17^), mainly due to cataract or small pupil size. A new trial (Clearsight) of TR screening is under way in Ontario that aims to address some of the problems of poor images by using nonmydriatic, ultra-wide-field imaging while retaining the advantages of nonmydriasis and patient convenience.^31^

We chose a handheld, nonmydriatic fundus camera (Smartscope PRO) for reasons of suitability in a diabetes clinic setting in India: cost, portability, and ease of training for non–eye care professionals. This camera has shown high sensitivity and specificity (>85%) in diagnosing DR compared with dilated fundus examination and even higher sensitivity and specificity in comparison with a 3-field view mydriatic table-top camera.^32,33^

### Limitations

The trial has limitations. It was relatively small (801 patients), although larger than most previous trials. We were unable to confirm the DR status of those who did not attend AEH, including 7 patients graded as having severe NPDR or worse by TR imaging. This lack of data means that many patients (4 with severe NPDR and 6 with PDR), even after being told of the possibility that they had DR, did not attend AEH, and warrants more effort in sensitizing patients about the visual implications.

## Conclusions

Our results suggest that TR is a more effective method leading to higher acceptance rates for ophthalmic examination for DR compared with UR. The benefits of TR may have been underestimated because the comparison group (UR) received more attention and individual referral compared with usual care in our setting. There is a need to address barriers, with special focus on older people, women, and those with no formal education, to enhance the uptake of DR screening. The advent of newer, better, and cheaper fundus cameras^34^ and emerging technologies, such as ultra-wide-field imaging,^31^ would make this approach more affordable and reliable. Our study was small and requires confirmation in a larger trial using different strategies of enhancing adherence.

## Notes

Supplement 1. Trial Protocol

Supplement 2. **eTable.** Hospital-Diagnosed Diabetic Retinopathy by Randomized Arm (Per Protocol Analysis) **eFigure.** Flowchart of Randomized Clinics

Supplement 3. Data Sharing Statement
