# Supplementary material for: Effectiveness of Teleretinal Imaging–Based Hospital Referral Compared With Universal Referral in Identifying Diabetic Retinopathy: A Cluster Randomized Clinical Trial
Source: JAMA Ophthalmol. 2019 May 9;137(7):786–92. doi: 10.1001/jamaophthalmol.2019.1070 (PMC6512266; doi:10.1001/jamaophthalmol.2019.1070)
Supplement: Supplement 1. — Trial Protocol [file jamaophthalmol-137-786-s001.pdf]

RESEARCH PROPOSAL FOR THE WELLCOME TRUST MASTERS FELLOWSHIP

Research Question:

What is the effectiveness of Telemedicine in identifying cases of Diabetic Retinopathy (DR) attending diabetes clinics compared with a universal hospital referral strategy?

Rationale

Diabetes Mellitus (DM) is a major health problem estimated to have affected 171 million people worldwide in 2000 and projected to rise to 366 million in 2030 [1]. India has the highest number of people with diabetes in the world with 31.7 million in 2000. This figure is estimated to rise to 79.4 million by 2030, based only on demographic changes, i.e. the growth of the older population where diabetes is more prevalent. However, lifestyle changes, especially increasing levels of obesity may lead to an even greater number of people with DM in India. Consequences of DM include Diabetic Retinopathy (DR) which is a major cause of avoidable blindness in both developing and the developed countries [2] and patients with DR are 25 times more likely to become blind than non-diabetics. Various studies conducted in India shows that 12 – 18% of the diabetic population develops DR [3-6]. In a population-based study conducted by Aravind Eye Hospitals in the Theni district of south India the prevalence of DM was found in 10.8 % of people aged 30 and over; of whom 12.2% had DR [4]. Prevalence rates of both DM and DR were higher in older people and in urban compared to rural areas.

Timely treatment can prevent vision loss due to diabetic retinopathy [7]. Treatment can yield substantial cost savings compared with the direct costs for those disabled by vision loss [8]. Though all diabetics require regular monitoring for diabetic retinopathy, only a very small proportion of the “known diabetics” receive a comprehensive eye examination [8]. Factors such as lack of access to eye hospitals, lack of ophthalmologists trained in diabetic retinopathy management and lack of awareness both by diabetic physicians, optometrists and patients. In particular, lack of knowledge among physicians and optometrists regarding diabetic retinopathy is highly unacceptable [9]. A study conducted on the awareness level of physicians in India reported that more than 67% of the physicians were unaware and about 10% were only partially aware of diabetic retinopathy [10]. When patients do attend ophthalmology clinics, it may be at a late stage of disease and their prognosis is poor. Treatment of end stage DR does not provide satisfactory results and is frustrating for both the patient and the ophthalmologist [3].

Population screening for diabetes and DR is costly and therefore is not appropriate as a regular screening strategy to identify DR. In the exiting context of healthcare, the critical challenges that need to be addressed include:

a. Identifying diabetics at risk of DR through targeted screening of known diabetic patients at diabetic/physician centres
b. Ensuring easy access to an ophthalmic centre for the diabetic patients who require further intervention
c. Enabling patients diagnosed with DR to comply with regular follow-up either at diabetic/physician centres or the ophthalmic centres, as appropriate

The conventional system is to refer all patients identified with diabetes to undergo an eye examination at an ophthalmic centre. However, only a very small percentage of the people referred in this mode actually present at the ophthalmic centre for an eye examination to confirm or rule out the presence of DR [11,12]. Thus opportunities for intervention and management of DR are lost.

Fundus examination is adequate for diagnosing DR and its severity. Technological advances in ophthalmic care have enabled the availability of digital imaging of fundus making it easier and more widely accessible [13]. Digital retinal photography without mydriasis by trained office staff has been reported as an effective mechanism to examine patients who are in need of diabetic retinopathy screening [14]. Use of telemedicine has been, in many instances, found to be valid and effective in achieving better outcome in the screening of, and interventions for, diabetes related complications as well as other systemic diseases [15-18]. Implementing retinal imaging technology in a primary care setting has been found to have resulted in a significant increase in rate of DR surveillance and in the rate of laser treatment for DR [11, 19]. But, there has not been an RCT to evaluate robustly the effectiveness of telemedicine in case finding of DR.

Coupling the recent advances in ophthalmology and information technology, Aravind Eye Hospitals has developed an innovative opportunistic case finding approach involving diabetes centres that binds digital retinal imaging with a robust IT solution - basically aimed at creating access for retinal screening. Telemedicine based digital retinal imaging for diabetic retinopathy has been installed in diabetes centres and the fundus images of suspected patients are sent to the Reading & Grading Centre at Aravind Eye Hospital where the images are graded using an indigenously developed software ADRES 4.0 (Aravind Diabetic Retinopathy Evaluation Software) and a report is sent back to the diabetes centre [20].

However, up-scaling this approach in India and other developing countries requires evidence about its effectiveness of achieving the desired objective. The proposed study in diabetes centres compares the effectiveness of telemedicine-based screening and hospital referral with that of a universal hospital referral strategy i.e. referring all eligible diabetic patients.

Objective: To compare the effectiveness of identifying Diabetic Retinopathy through referral followed by telemedicine based digital retinal imaging in Diabetes Centres with that of the a universal referral system

Hypothesis: Telemedicine based digital retinal imaging in a diabetes centre will identify proportionately more diabetic patients with DR and be associated with higher acceptance rates compared to Universal referral.

Study Plan:

Design: The design is a cluster randomised trial (RCT). Diabetes centres will be randomized to one of two strategies: telemedicine referral (TR) or universal referral (UR). Patients attending diabetes centres within a distance of 10 kms from Aravind Eye Hospital Madurai (i.e. within the urban and peri-urban area) will undergo one of these randomized interventions. A cluster RCT is required to avoid patients in the same centre being offered different screening interventions which might present both logistic and ethical difficulties.

Procedures: All patients identified in either arm meeting the inclusion and exclusion criteria will be referred to the clinic study coordinator who will seek fully informed patient consent (see below, under Ethical considerations) prior to enrolling the patient. Those enrolled will be counselled about DM and the importance of vision screening, and will be invited for either digital fundus imaging at the diabetes centre (TR) or for detailed examination for diabetic retinopathy (DR) at an eye hospital (Aravind Eye Hospital Madurai, AEH). Randomisation is at the level of the centre and there is no individual patient randomisation. In TR the diabetes centre will be equipped with a digital imaging system for screening the fundus and the patients diagnosed with DR or probable DR will be counselled to visit AEH for a full retinal examination to confirm (or not) the presence of

DR. In UR, the eligible patients will be counselled and referred to undergo eye examination at AEH. In both the arms the counselling and the awareness creation materials will be standardised.

A total of eight diabetes centres will be identified and four each will be randomly allocated to each arm stratified by distance from Aravind Eye Hospital Madurai. The aim of stratification is to ensure that there is no imbalance in the randomized arms in proximity to the hospital. The stratification criteria will be <5km, ≥ 5-10km.This covers the most densely populated areas (less than 5km), and the outer peri-urban/rural areas. An interviewer cum counsellor will be appointed at each of the diabetes centres in UR who will administer the consent form, collect patient data and do the referral. In the diabetes centres in TR, the interviewer cum counsellor will also be trained in fundus photography through a structured two-weeks training. The study will be carried out for a period of six months and a window of one month after referral will be given for the patient to visit the eye hospital.

At the end of the one month window after the last referral, those patients who have not complied with the referral will be contacted. A short semi structured interview will be conducted with (i) all those who decline referral and/or imaging to understand the barriers to compliance (ii) a sample of those who do attend Aravind Eye Hospital to understand the drivers of acceptance. The topic guide for the interviews will be developed from previous literature and other studies on barriers and drivers to cataract surgery uptake undertaken by Aravind Eye Hospital [21]. The results from these interviews will form a part of the final overall evaluation of the trial by adding a qualitative component. The interviewer will also ask about any other eye examinations that have been conducted by other eye professionals (ophthalmologists or optometrists) since patients may have chosen to attend elsewhere for the eye examination. In this situation information will be sought on the DR findings.

Diabetic Retinopathy:
DR will be graded using the standard classification (from reference [8])

DIABETIC RETINOPATHY DISEASE SEVERITY SCALE

**Disease Severity Level**	**Findings Observable upon Dilated Ophthalmoscopy**
No apparent retinopathy	No abnormalities
Mild NPDR	Microaneurysms only
Moderate NPDR	More than just microaneurysms but less than severe NPDR
Severe NPDR	Any of the following (4-2-1 rule) and no signs of proliferative retinopathy:<br>Severe intraretinal hemorrhages and microaneurysms in each of four quadrants<br>Definite venous beading in two or more quadrants<br>Moderate IRMA in one or more quadrants
PDR	One or both of the following:<br>Neovascularization<br>Vitreous/preretinal hemorrhage

IRMA = intraretinal microvascular abnormalities; NPDR = nonproliferative diabetic retinopathy; PDR = proliferative diabetic retinopathy

Data collection: A database will be designed to capture:

1. Basic details from the eligible patients such as name, age, contact information, blood sugar level, HBA1C, duration of diabetes, date of diagnoses, other vascular complications etc.
2. Details of the fundus examination also will be recorded for participants from both the arms
3. Responses to interview that probe barriers to non-response and drivers of acceptance

Sample size estimates:
The following preliminary assumptions were made:

(i) The proportion of patients in UR diagnosed with DR at AEH is 30% of the referred patients
(ii) The proportion of patients in TR diagnosed with DR at AEH is 50% of the referred patients
(iii) Power of 90% and alpha of 0.01 for a 2 sided test
(iv) The design effect is 2 (derived from reference [4])

The estimated total sample size is 616 patients, equally distributed in each arm. Each of the 8 centres will recruit between 80 and 100 patients eligible for referral to AEH. We anticipate the time taken to recruit this number of eligible patients will be approximately 6 months. This is a conservative estimate because it is likely that a substantial number of eligible patients will be available. Population estimates show that the proportion with DR in diabetics is 12% and is higher in urban areas, in high income groups and in older people (50+) and with longer duration of diabetes [5].

Inclusion and Exclusion Criteria:

Inclusion criteria: Diabetes patients (diagnosed with diabetes as designated by International Classification of Diseases (ICD-9 code 250) in the age group of ≥50 years

Exclusion criteria:

a. Patients <50 years of age
b. Distance from residence to AEH beyond 25km
c. Already enrolled as study patients at the diabetes centre
d. Patients screened for DR in free camps organized by the diabetes centre
e. Diabetic patients who underwent retinal examination in the previous year prior to this intervention
f. Patients with disability (physical or mental) who have difficulty in travelling to the eye hospital

Data preparation and analysis

Data checking

Data collected using the structured questionnaire forms will be entered into a custom designed data base (MS Access-2013) using double data entry. Comparison and consistency checks will be performed using Epi-info software (version: 3.4.3). The final data set will be transferred to STATA 14 (StataCorp Texas) for further cleaning and management. Variables such as age, annual income, education, distance, duration of diabetes etc. will be categorised appropriately. The paper forms will be scanned and archived for further references.

Outcomes

The primary outcome will be diabetic retinopathy (DR) confirmed by dilated fundus examination at the hospital.
The secondary outcome will be hospital attendance.

Strategies of analysis

We will report two strategies of analysis for the primary outcome.
The primary intention to treat (ITT) analysis will include all patients eligible for referral in each randomised arm irrespective of compliance with referral. Eligibility for referral is defined according to the protocol, i.e. those who were offered a hospital referral. In the UR arm all patients will be offered a hospital referral. In the TR arm only those identified by teleretinal imaging as requiring further investigation or with inadequate images will be referred.

The secondary per-protocol (PP) analysis will include only patients who complied with referral (defined as attendance at Aravind Eye Hospital or at other eye specialist).

Statistical analysis

The data will be analysed using Stata14 (StataCorp Texas). All analyses will take account of the cluster trial design, i.e. the clinic/centre is the unit of randomization. The survey suite of commands ("svy") in Stata will be used for all regression models and cross tabulations.

We will first compare the distribution of characteristics between the trial arms including important prognostic covariates such as age, sex, duration of diabetes. The primary outcome will be analysed using Poisson regression comparing the proportion of DR in the TR arm with that in UR and report the risk ratio (RR) and 95% Confidence Interval (CI) in unadjusted analysis and covariate adjusted analysis. These analyses will be undertaken for the ITT and PP populations. The ITT primary outcome analysis will represent the trial main result.

For the secondary outcome we will investigate characteristics associated with hospital attendance using Poisson regression in a model including randomised arm. We will first check whether characteristics of attendance vary by randomised arm in separate analyses before combining in a single model.

We will also report descriptive analyses based on the semi structured interviews. These will be transcribed into a data base and text analysis techniques will be used to group the responses (this may necessitate translation into English if Tamil software is not available).

Ethical consideration: The protocol including the procedures for fully informed consent will be presented for approval from the Research Committee and the Institutional Review Board of Aravind Eye Hospital, India. Eligible participants in the telemedicine arm will be given an information sheet in the local language which will explain the purpose of the study and what

procedures are involved and asked for consent to access their clinical records. Patients in the usual care arm will be asked for consent to access their medical records. In both arms patients will be told that they may in future be interviewed for their views about diabetes and DR. Patients will be reassured that their personal and clinical details will be kept confidential and in the telemedicine arm that their usual care will not be affected if they choose not to participate For patients who are illiterate the information will be read out to them. All participants will be given the information sheet to take home and the contact number of the study co-ordinator. At the next visit of the participant the centre co-ordinator will answer any further questions from the patient and seek written consent (or for illiterate patients, a thumb impression will be taken)

Benefits of study: The vision loss due to DR cannot be reversed and hence timely detection of the problem is crucial in preventing advancement of the disease there by saving the vision. This will be possible only by encouraging the patients at risk to undergo fundus examination on a regular basis. Very low rate of detection is the most important challenge in the current scenario. The findings of this study will provide evidence as to which method of case finding is more effective in promoting early detection of DR. The results can be used to promote best practices among the providers and to create awareness in the community to achieve effective management of DR. However, no RCT on telemedicine in DR has been done in India or (as far as we know) in any other country. If telemedicine for DR is effective in the urban/ semi urban environment we will conduct a similar trial in the future in the rural setting.

References

1. Wild S, Roglic G, Green A, Sicree R, King H. Global Prevalence of Diabetes: Estimates for the year 2000 and projections for 2030. Diabetes Care. 2004; 27:1047-1053.
2. Recommendations in Prevention of Blindness from Diabetes Mellitus: report of a WHO consultation. Geneva, Switzerland 2005 World Health Organization
3. Rani, PK, Raman R, Sharma V et.al.; Analysis of a comprehensive diabetic retinopathy screening model for rural and urban diabetics in developing countries; Br J Ophthalmol 2007;91:1425–1429.
4. Narendran V, John RK, Raghuram A, Ravindran RD Nirmalan PK,Thulasiraj RD. Diabetic retinopathy among self reported diabetics in southern India: a population based assessment. Br J Ophthalmol.2002; 86:1014–1018.
5. Namperumalsamy P, Kim R, Vignesh TP, Nithya N, Royes J, Gijo T, Thulasiraj RD, Vijayakumar V. Prevalence and risk factors for diabetic retinopathy: a population-based assessment from Theni District, south India. Br J Ophthalmol. 2009;93:429-34.
6. Rema M, Premkumar S, Anitha B, Deepa R, Pradeepa R, Mohan V. Prevalence of diabetic retinopathy in urban India: the Chennai Urban Rural Epidemiology Study (CURES) eye study Invest Ophthalmol Vis Sci 2005; 46 : 2328-33.
7. Early Treatment Diabetic Retinopathy Study Research Group. Early photocoagulation for diabetic retinopathy. ETDRS report number 9. Ophthalmology 1991;98:766-85
8. American Academy of Ophthalmology Retina Panel: Diabetic Retinopathy preferred practice pattern guidelines. San Francisco CA: American Academy of Ophthalmology,2008
9. Ghosh S, Mukhopadhyay S, Maji D, Halder D. Awareness of diabetic retinopathy among physicians and optometrists in a district of West Bengal. Indian J Public Health.2007;51:228-30.
10. Choudhary H, Choudhury H, Biswas T; Diabetic Retinopathy: How Aware are the Physicians?; AIOC 2009 Proceedings
11. Taylor CR,, Merin LM, Salunga AM et al.; Improving Diabetic Retinopathy Screening Ratios Using Telemedicine-Based Digital Retinal Imaging Technology-The Vine Hill Study; Diabetes Care 2007;30:574-578
12. Mumba M, Hall A, Lewallen S; Compliance with eye screening examinations among diabetic patients at a Tanzanian referral hospital; Ophthalmic Epidemiol. 2007;14:306-10.
13. Williams GA, Scott IU, Haller JA, Maguire AM, Marcus D Mc Donald R.; Single field fundus photography for diabetic retinopathy screening. A Report by American Academy of Ophthalmology. Ophthalmology 2004;111:1055-1062
14. Massaro L, Curry WJ, Quillen D; Screening for Diabetic Retinopathy: Perceived Barriers and Patient Acceptability of Digital Scans. JCOM 2010;17:401-405
15. Weinstock R, Brooks G, Palmas W et al. Lessened decline in physical activity and impairment of older adults with diabetes with telemedicine and pedometer use: results from the IDEATel study. Age and Ageing 2011;40:105–111
16. Liesenfeld B, Kohner E, Piehlmeirer W et al. A Telemedical Approach to the Screening of Diabetic Retinopathy: Digital Fundus Phtography. Diabetes Care2000; 23: 345-348
17. Meyer B, Raman R, Hemmen T.Efficacy of site-independent telemedicine in the STRokE DOC trial: a randomised, blinded, prospective study. Lancet Neurol.2008;7:787–795
18. Wallace P, Haines A, Harrison R; Joint tele-consultations (virtual outreach) versus standard outpatient appointments for patients referred by their general practitioner for a specialist opinion: a randomised trial. Lancet 2002; 359; 1961-1968
19. Singh R, Ramasamy K, Abraham C, Gupta V, Gupta A. Diabetic retinopathy: An update. Indian J Ophthalmol 2008;56:179-88

20. Namperumalsamy P, Prasad NM, Shankar S, Kim R; Software for Reading and Grading Diabetic Retinopathy; ADRES 3.0. Diabetes Care 2007;30: 2302-2306
21. Fletcher AE, Donoghue M, Devavaram J, Thulasiraj RD, Scott S, Abdalla M, Shanmugham C.A.K, Bala Murugan. Low uptake of eye services in rural India; Archives Ophthalmology 1999;117:1393-1399

Effectiveness of Teleretinal ImagingCompared with Universal Referral in Identifying Diabetic Retinopathy

Data preparation

Data collected using the structured questionnaire forms will be entered into a custom designed data base (MS Access-2013) using double data entry. Comparison and consistency checks will be performed using Epi-info software (version: 3.4.3). The final data set will be transferred to STATA 14 (StataCorp Texas) for further cleaning and management. Variables such as age, annual income, education, distance, duration of diabetes etc. will be categorised appropriately. The paper forms will be scanned and archived for further references.

Statistical Plan

Outcomes

The primary outcome will be diabetic retinopathy (DR) confirmed by dilated fundus examination at the hospital.

The secondary outcome will be hospital attendance.

Strategies of analysis

We will report two strategies of analysis for the primary outcome.

The primary intention to treat (ITT) analysis will include all patients eligible for referral in each randomised arm irrespective of compliance with referral. Eligibility for referral is defined according to the protocol, i.e. those who were offered a hospital referral. In the UR arm all patients will be offered a hospital referral. In the TR arm only those identified by teleretinal imaging as requiring further investigation or with inadequate images will be referred.

The secondary per-protocol (PP) analysis will include only patients who complied with referral (defined as attendance at Aravind Eye Hospital or at other eye specialist).

Statistical analysis

The data will be analysed using Stata14 (StataCorp Texas). All analyses will take account of the cluster trial design, i.e. the clinic/centre is the unit of randomization. The survey suite of commands (“svy”) in Stata will be used for all regression models and cross tabulations.

We will first compare the distribution of characteristics between the trial arms including important prognostic covariates such as age, sex, duration of diabetes. The primary outcome will be analysed using Poisson regression comparing the proportion of DR in the TR arm with that in UR and report the risk ratio (RR) and 95% Confidence Interval (CI) in unadjusted analysis and covariate adjusted analysis. These analyses will be undertaken for the ITT and PP populations. The ITT primary outcome analysis will represent the trial main result.

For the secondary outcome we will investigate characteristics associated with hospital attendance using Poisson regression in a model including randomised arm. We will first check whether characteristics of attendance vary by randomised arm in separate analyses before combining in a single model.
