# Supplementary material for: Effectiveness of Teleretinal Imaging–Based Hospital Referral Compared With Universal Referral in Identifying Diabetic Retinopathy: A Cluster Randomized Clinical Trial
Source: JAMA Ophthalmol. 2019 May 9;137(7):786–92. doi: 10.1001/jamaophthalmol.2019.1070 (PMC6512266; doi:10.1001/jamaophthalmol.2019.1070)
Supplement: Supplement 2. — eTable. Hospital-Diagnosed Diabetic Retinopathy by Randomized Arm (Per Protocol Analysis) eFigure. Flowchart of Randomized Clinics [file jamaophthalmol-137-786-s002.pdf]

Supplementary Online Content

Joseph S, Kim R, Ravindran RD, Fletcher AE, Ravilla TD. Effectiveness of teleretinal imaging–based hospital referral compared with universal referral in identifying diabetic retinopathy: a cluster randomized clinical trial. *JAMA Ophthalmol*. Published online May 9, 2019. doi:10.1001/jamaophthalmol.2019.1070

eTable. Hospital-Diagnosed Diabetic Retinopathy by Randomized Arm (Per Protocol Analysis)

eFigure. Flow Chart of Randomized Clinics

This supplementary material has been provided by the authors to give readers additional information about their work.

© 2019 Joseph S et al. *JAMA Ophthalmology*.

eTable. Hospital-Diagnosed Diabetic Retinopathy by Randomized Arm (Per Protocol Analysis[1])

Diabetic retinopathy [a]	Risk ratio[b]	95% CI[b]	*p*-value[b]
TR vs UR [c]	2.00	1.30, 3.07	.002
TR vs UR [d]	1.75	1.12, 2.74	.014
Age (years)	0.99	0.95, 1.02	.42
Women	1.05	0.67, 1.65	.82
Blood Sugar (mg/100ml)	1.00	0.99, 1.00	.91
Diabetes Duration (years)	1.05	1.02,1.09	.003
On Insulin Treatment	1.38	0.87,2.18	.17
Cardio-vascular Disease	0.75	0.27, 2.07	.58
Hypertension	0.91	0.56, 1.49	.71

[1] all patients referred to Hospital in each randomised arm who complied with referral
[a] Hospital diagnosed Diabetic Retinopathy
[b] Risk ratio, 95% Confidence Interval and p value from Poisson model
[c] Teleretinal imaging and Referral (TR) compared to Universal Referral, unadjusted for other covariates
[d] Teleretinal imaging and Referral (TR) compared to Universal Referral, adjusted for other covariates

© 2019 Joseph S et al. *JAMA Ophthalmology*.

eFigure. Flow Chart of Randomized Clinics

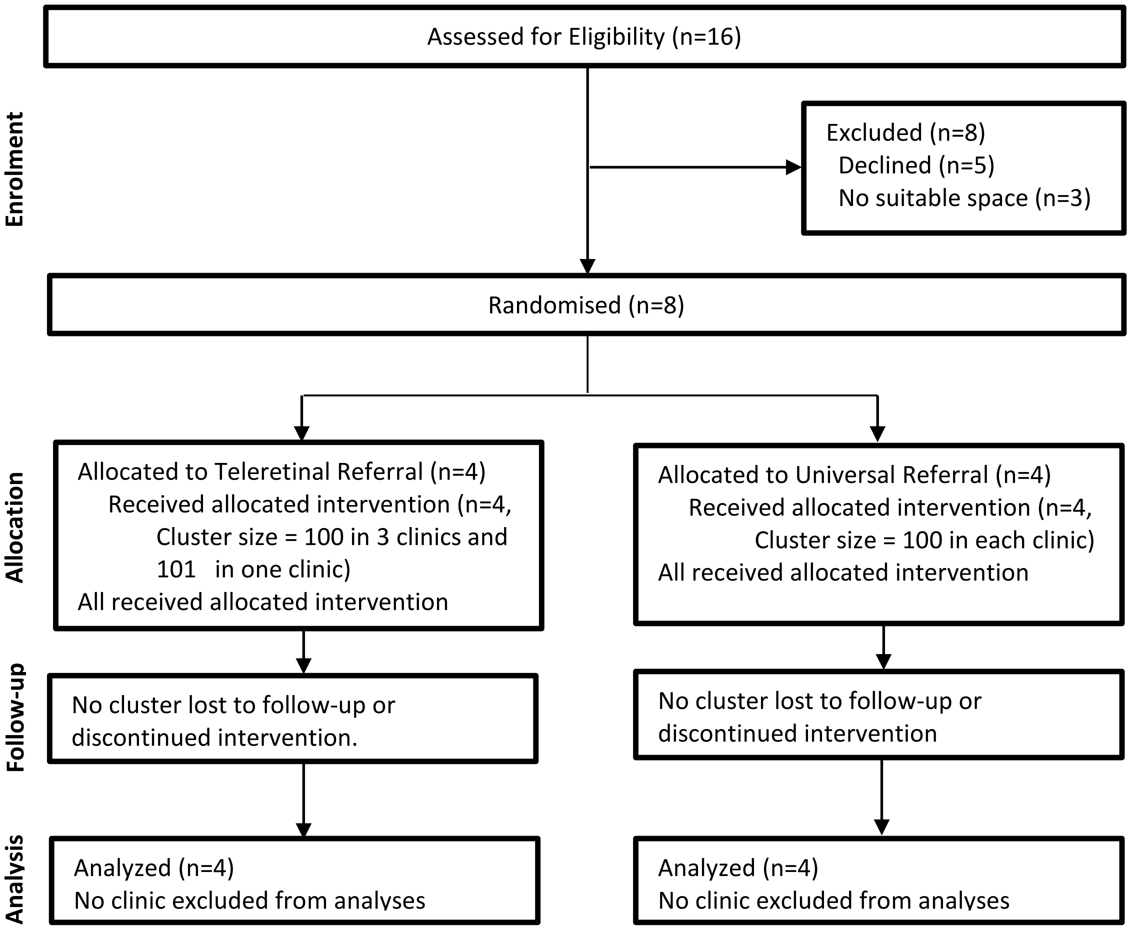

© 2019 Joseph S et al. *JAMA Ophthalmology*.
