# Supplementary material for: Effectiveness of Teleretinal Imaging–Based Hospital Referral Compared With Universal Referral in Identifying Diabetic Retinopathy: A Cluster Randomized Clinical Trial
Source: JAMA Ophthalmol. 2019 May 9;137(7):786–92. doi: 10.1001/jamaophthalmol.2019.1070 (PMC6512266; doi:10.1001/jamaophthalmol.2019.1070)
Supplement: Supplement 3. — Data Sharing Statement [file jamaophthalmol-137-786-s003.pdf]

Data Sharing Statement

Joseph. Effectiveness of Teleretinal Imaging-Based Hospital Referral Compared With Universal Referral in Identifying Diabetic Retinopathy. *JAMA Ophthalmol*. Published May 09, 2019.
10.1001/jamaophthalmol.2019.1070

Data
Data available: No
